# Pathogenesis of bloodstream infection in children with blood cancer

**DOI:** 10.3892/etm.2012.738

**Published:** 2012-10-08

**Authors:** HUA LV, BOTAO NING

**Affiliations:** 1 Departments of Infection Control and; 2 Pediatric ICU, Children’s Hospital, School of Medicine, Zhejiang University, Hangzhou, Zhejiang 310003, P.R. China

**Keywords:** bloodstream infection, pathogen, drug resistance

## Abstract

The aim of the present study was to characterize the distribution and antibiotic resistance of pathogens isolated from patients with bloodstream infections (BSIs) in the Hematology and Oncology department of the Affiliated Children’s Hospital of Zhejiang University Medical School (Hangzhou, China), between January and December 2010 and to provide early and appropriate support for the clinical administration of antibiotics. Out of 1,500 inpatients, 161 children who were diagnosed with BSI based on the national diagnostic criteria were retrospectively analyzed. Neutropenia was defined as an absolute neutrophil count (ANC) in the peripheral blood of less than 0.5×10^9^ cells/l. A microbiologically documented infection (MDI) was defined as when the causative pathogen was isolated from the blood. Drug susceptibility tests were performed using a VITEK-60 AutoMicrobic System and the Kirby-Bauer disk diffusion method. The data were analyzed using STATA software (version 9.0) and a two-sided P-value of ≤0.05 was considered to indicate a statistically significant difference. A total of 79 strains were isolated from the blood specimens. The incidence of BSI was 10.73% (161/1,500). Gram-positive cocci, Gram-negative bacilli and fungi accounted for 55.70, 43.04 and 1.27% of the BSIs, respectively. *Staphylococcus epidermidis* (20.25%), *Escherichia coli* (15.19%) and *Klebsiella pneumoniae* (15.19%) were frequently identified isolates. The staphylococci were susceptible to vancomycin and linezolid, while *Escherichia coli* and *Klebsiella pneumoniae* were sensitive to cefoperazone/sulbactam, imipenem and meropenem. In conclusion, Gram-positive bacteria are slightly more prevalent than Gram-negative bacteria in BSI and the selection of antibiotics according to the susceptibility test results is superior to empirical treatment. It is essential to administer antimicrobial agents early and appropriately to treat child blood cancer patients with BSI.

## Introduction

Sepsis and bacteremia are referred to as bloodstream infection (BSI) (1). Children with blood cancer often suffer from neutropenia, immune suppression and damage to the skin, oral cavity and mucous membrane of the digestive tract due to cancer, chemotherapy, radiotherapy and the administration of immunosuppressants. This allows invasive and conditional bacteria to invade the bloodstream through the mucous membrane barrier (2). In order to elucidate the pathogenic distribution of BSI and provide strategies for its prevention and treatment, an investigation of BSI in patients with blood cancer was performed at the Affiliated Children’s Hospital of Zhejiang University Medical School (Hangzhou, China) between January and December 2010.

## Patients and methods

### Definitions

Neutropenia was defined as an absolute neutrophil count (ANC) in the peripheral blood of <0.5×10^9^ cells/l (3,4). Febrile patients were defined as those yielding 1 axillary temperature measurement of ≥38.5°C or 2 measurements of 38–38.4°C within a 4-h interval (4). BSI was diagnosed according to the infection diagnostic standards published by the Medical Administration Department of the Chinese Health Ministry in 2001 (5). A clinical diagnosis was defined as when temperature was >38°C or <36°C and occurred with one of the following conditions: i) invasion or migration lesions; ii) systemic poisoning symptoms without an apparent focal infection; iii) unexplained skin rash, petechia, hepatosplenomegaly or increased neutrophil count with a left shift; iv) systolic blood pressure <12 kPa (90 mmHg) or a blood pressure decrease of >5.3 kPa (40 mmHg) compared with the original systolic pressure. An etiological diagnosis was defined according to the clinical diagnosis and the occurrence of one of the following events: i) a pathogenic microorganism was cultured from the blood; ii) a pathogen antigen was detected in the blood.

### Patients

At the Affiliated Children’s Hospital of Zhejiang University Medical School between January and December 2010, 1,500 new patients were admitted to the hematology department. Of these, 161 patients (93 males and 68 females) with a median age of 7 years (range, 1 month to 17 years) developed BSI. The basic diseases of the BSI patients consisted of 112 cases of acute lymphocytic leukemia, 34 of acute myelogenous leukemia, 10 of lymphoma, 4 of hemophagocytic syndrome and 1 of Langerhans cell histiocytosis. The etiologically diagnosed patients with positive blood culture were categorized as the microbiologically documented infection (MDI) group and the clinically diagnosed patients with negative blood culture were the non-MDI group (6,7). The study was approved by the ethics committee of the Children’s Hospital affiliated to the School of Medicine of Zhejiang University, and written, informed consent was obtained from the patients’ parents or legal guardians in all cases.

### Instruments and reagents

Bacteria were identified using a VITEK-60 AutoMicrobic System purchased from the Marcel Mérieux Company, Lyon, France. Susceptibility testing paper and susceptibility medium (MH medium) were purchased from the Oxiod Ltd. (Basingstoke, UK) and Marcel Mérieux, respectively.

### Antibiotic susceptibility test

A total of 161 blood samples were cultured and routine drug susceptibility tests were performed using the VITEK-60 AutoMicrobic System and the Kirby-Bauer disk diffusion method. The extended-spectrum β-lactamase (ESBL) strains were detected using a double disk test. The drug resistance criteria followed the Clinical Laboratory Standards Institute (CLSI) guidelines set in 2008 (8). If more than 2 consecutive results were the same in one patient, the results of the blood culture were recorded once; if the results differed, each result of the blood culture was recorded.

### Statistical analysis

The data were analyzed using STATA software, version 9.0 (StataCorp LP, College Station, TX, USA). A two-sided P-value of ≤0.05 was considered to indicate a statistically significant difference.

## Results

### Incidence of BSI and rate of MDI

Out of 1,500 child patients, 161 were diagnosed as having BSI. The incidence was 10.73% (161/1,500) and 79 of the blood cultures were positive so the positive rate of the blood culture was 49.07% (79/161).

### Distribution of pathogens

A total 79 strains were detected in 161 patients. Gram-positive bacteria, Gram-negative bacteria and fungi accounted for 55.70 (44/79), 43.04 (34/79) and 1.27% (1/79) of the BSIs, respectively. The most common pathogens were *Staphylococcus epidermidis*, *Escherichia coli* and *Klebsiella pneumoniae* which accounted for 20.25, 15.19 and 15.19%, respectively. The distribution of pathogens is shown in Table I.

### Drug resistance results of common pathogens

A total of 27 β-lactamase-positive strains were detected from the 31 identified staphylococci, accounting for 87.1% (27/31). A total of 16 *Staphylococcus epidermidis* strains exhibited 100% resistance to oxacillin, 72.73% of the 11 strains of other coagulase-negative staphylococci (CoNS) were resistant to oxacillin and the 4 strains of *Staphylococcus aureus* were not resistant to oxacillin. A total of 9 ESBL-producing strains were detected from 12 strains of *Escherichia coli* (75%) and 5 ESBL-producing strains were detected from 12 strains of *Klebsiella pneumoniae* (41.7%). The staphylococcus and Gram-negative bacteria resistance rates are shown in Tables II and III, respectively.

### Treatment and outcome

All the 161 BSI patients were treated with antimicrobial agents, which included β-lactams, carbapenems, glycopeptides, nitroimidazoles and anti-fungals. A total of 30 patients (18.63%) received single drugs, 63 patients (39.13%) received 2 drugs and 68 patients (42.24%) received 3 or more drugs. The 5 most commonly used antimicrobial agents were vancomycin, imipenem, cefoperazone/sulbactam, piperacillin/tazobactam and meropenem. The mean treatment times were was 10.77±4.88 days for the non-MDI group and 8.27±2.85 days for the MDI group (t=3.81, P=0.0002). Granulocyte colony-stimulating factor (G-CSF) and venous injections of immunoglobulin were administered to the patients with neutropenia at the same time as the antimicrobial therapy. With regard to the fungal infection, oral fluconazole was administered for 3 days while ANC was <1.0×10^9^ cells/l. Of the 161 patients, 156 were successfully cured and 5 BSI patients succumbed to uncontrolled systemic infection. One of these patients was infected with *Pseudomonas aeruginosa*, 2 were infected with *Klebsiella pneumoniae* and 2 were negative in the blood culture. The mortality rate was 3.1% (5/161).

## Discussion

The blood cancer patients were susceptible to infection complications, particularly bacterial sepsis, as well as low immune function caused by malignant tumors and the impairment of physiological barrier defense function causing topical edema, erosion, necrosis, compression and obstruction. Neutropenia resulting from chemotherapy and/or radiotherapy may be the single most important risk factor responsible for the sepsis (9). The data revealed that the incidence of BSI was 10.73% (161/1,500) versus 5.2–18.2% as reported previously (10,11). The positive blood culture rate of 49.07% in BSI patients was lower than that of other studies (12,13). This may be since we did not routinely collect double blood samples from various sites simultaneously.

Our study revealed that the distribution of Gram-positive bacteria, Gram-negative bacteria and fungi was 55.70, 43.04 and 1.27%, respectively. The 3 most common pathogens were *Staphylococcus epidermis*, *Escherichia coli* and *Klebsiella pneumoniae*. Celkan *et al*(14) reported that 60% of the pathogens separated from the blood culture of 159 oncological patients were Gram-positive bacteria and the CoNS was the dominant. These results were similar to those of the present study. Gram-positive bacterial infection is an increasing trend in cancer patients and the infection rates of CoNS and streptococci have increased the most rapidly (15). This may contribute to the extremely high usage levels of broad spectrum antimicrobial agents for use against Gram-negative bacteria and invasive surgery, such as deep vein catheter implantation. Long-term and extremely high levels of broad spectrum antibiotic use may potentially cause the over-proliferation of Gram-positive bacteria and catheter puncture may result in mucocutaneous injury, making conditional pathogenic infection possible. The most common Gram-positive bacteria (staphylococci) exhibited no or low drug resistance to vancomycin, linezolid and moxifloxacin, while the dominant Gram-negative bacteria had no or low drug resistance to cefoxitin, imipenem and piperacillin/tazobactam. According to the guidelines of the American Infectious Disease Association (16,17), as well as the supportive treatment, antibiotics were administered early, at adequate levels and in combinations such as imipenem with vancomycin or piperacillin/tazobactam with vancomycin. As soon as the results of the antibiotic susceptibility tests were available, the antibiotics were adjusted accordingly. The mean treatment time of the MDI group was less than that of the non-MDI group and this difference was observed to be statistically significant (t=3.81, P=0.0002). This demonstrated that antibiotic selection according to the susceptibility test results was superior to empirical treatment during the whole of the anti-infectious treatment, although the latter was important in the initial period. Of the 5 patients who succumbed to infection, 3 were infected with Gram-negative bacteria and no bacteria were cultured in the remaining 2. Although the mortality rate was low (3.1%, 5/161), certain patients exhibited negative pathogenic cultures.

Although the fungal infection accounted for 1.27% (1/161) of the data, various pathogens, such as fungi, viruses and parasites, may infect patients with neutropenia and fungemia may be the prime cause of mortality (18). Mor *et al*(19) reported that 7.2% of the 1,047 children hospitalized in a Hematology/Oncology department were diagnosed with a proven invasive fungal infection. The low detection rate of invasive fungal infection in the present study may be due to the difficulty of culturing fungi. The lack of sensitive and specific detection methods possibly contributed to the prophylactic usage of oral fluconazole when the ANC was less than 1.0×10^9^ cells/l.

In conclusion, Gram-positive bacteria are the dominant pathogens in child blood cancer patients with BSI. Antibiotic selection according to the susceptibility test results is superior to empirical treatment. Antibiotic susceptibility tests are extremely important for the drug adjustment. In order to decrease the incidence and improve the outcome of BSI, improved prevention, surveillance of antibiotic resistance, early detection and advanced therapeutic strategies should be focused on.

## Figures and Tables

**Table I:** Distribution of BSI pathogens.

Pathogens	Number of strains	Proportion (%)
Gram-positive bacteria	44	55.70
*Staphylococcus epidermidis*	16	20.25
Other CoNS	11	13.92
Streptococcus	6	7.59
*Staphylococcus aureus*	4	5.06
Enterococcus	4	5.06
Micrococcus	2	2.53
Bacillus	1	1.27
Gram-negative bacteria	34	43.04
*Escherichia coli*	12	15.19
*Klebsiella pneumoniae*	12	15.19
*Pseudomonas aeruginosa*	5	6.33
*Sphingomonas paucimobilis*	2	2.53
*Aeromonas hydrophila*	2	2.53
*Salmonella thompsoni*	1	1.27
Fungus	1	1.27
*Candida albicans*	1	1.27
Total	79	100.00

BSI, blood stream infection; CoNS, coagulase-negative staphylococcus.

**Table II:** Drug resistance pattern of staphylococci isolated from the blood of patients with BSI.

	Drug resistance rate (%)		
Drugs	*Staphylococcus epidermidis* (n=16)	Other CoNS (n=11)	*Staphylococcus aureus* (n=4)
Clindamycin	50.00	45.45	50.00
Linezolid	0.00	0.00	0.00
Ampicillin/sulbactam	100.00	72.73	0.00
Gentamicin	37.50	27.27	25.00
Oxacillin	100.00	72.73	0.00
Rifampicin	12.50	9.09	0.00
Sulfamethoxazole	93.75	36.36	75.00
Vancomycin	0.00	0.00	0.00
Moxifloxacin	0.00	0.00	0.00
Erythromycin	93.75	54.55	75.00
Furantoin	0.00	0.00	0.00
Levofloxacin	0.00	27.27	25.00
Penicillin-G	100.00	81.82	75.00
Tetracycline	25.00	9.09	0.00

BSI, blood stream infection.

**Table III:** Drug resistance pattern of common Gram-negative bacteria isolated from the blood of patients with BSI.

	Drug resistance rate (%)		
Drugs	*Escherichia coli* (n=12)	*Klebsiella pneumoniae* (n=12)	*Pseudomonas aeruginosa* (n=5)
Cefoperazone/sulbactam	0.00	0.00	100.00
Cephazoline	83.33	50.00	100.00
Cefoxitin	0.00	0.00	0.00
Amikacin	8.33	0.00	0.00
Levofloxacin	33.33	16.67	100.00
Cefuroxime	83.33	58.33	0.00
Ceftazidime	75.00	50.00	100.00
Ampicillin	100.00	83.33	0.00
Cefpiramide	75.00	50.00	0.00
Gentamicin	83.33	50.00	0.00
Imipenem	0.00	0.00	0.00
Meropenem	0.00	0.00	80.00
Cefotaxime	75.00	41.67	0.00
Piperacillin/tazobactam	0.00	25.00	0.00

BSI, blood stream infection.
